# Dual Action of Zn^2+^ on the Transport Cycle of the Dopamine Transporter*

**DOI:** 10.1074/jbc.M115.688275

**Published:** 2015-10-26

**Authors:** Yang Li, Peter S. Hasenhuetl, Klaus Schicker, Harald H. Sitte, Michael Freissmuth, Walter Sandtner

**Affiliations:** From the Institute of Pharmacology, Center of Physiology and Pharmacology, Medical University of Vienna, Waehringerstrasse 13a, A-1090 Vienna, Austria

**Keywords:** dopamine, dopamine transporter, electrophysiology, kinetics, neurotransmitter release, neurotransmitter transport

## Abstract

The dopamine transporter shapes dopaminergic neurotransmission by clearing extracellular dopamine and by replenishing vesicular stores. The dopamine transporter carries an endogenous binding site for Zn^2+^, but the nature of the Zn^2+^-dependent modulation has remained elusive: both, inhibition and stimulation of DAT have been reported. Here, we exploited the high time resolution of patch-clamp recordings to examine the effects of Zn^2+^ on the transport cycle of DAT: we recorded peak currents associated with substrate translocation and steady-state currents reflecting the forward transport mode of DAT. Zn^2+^ depressed the peak current but enhanced the steady-state current through DAT. The parsimonious explanation is preferential binding of Zn^2+^ to the outward facing conformation of DAT, which allows for an allosteric activation of DAT, in both, the forward transport mode and substrate exchange mode. We directly confirmed that Zn^2+^ dissociated more rapidly from the inward- than from the outward-facing state of DAT. Finally, we formulated a kinetic model for the action of Zn^2+^ on DAT that emulated all current experimental observations and accounted for all previous (in part contradictory) findings. Importantly, the model predicts that the intracellular Na^+^ concentration determines whether substrate uptake by DAT is stimulated or inhibited by Zn^2+^. This prediction was directly verified. The mechanistic framework provided by the current model is of relevance for the rational design of allosteric activators of DAT. These are of interest for treating *de novo* loss-of-function mutations of DAT associated with neuropsychiatric disorders such as attention deficit hyperactivity disorder (ADHD).

## Teaser

**Background:** Zn^2+^ was originally proposed to inhibit dopamine uptake by the dopamine transporter (DAT).

**Results:** Zn^2+^ depressed peak currents, and modulated steady state-currents through DAT in a manner dependent on internal Na^+^.

**Conclusion:** At physiological intracellular Na^+^, Zn^2+^ activates both the forward and exchange transport mode of DAT.

**Significance:** The action of Zn^2+^ is of interest for rescuing conformationally trapped DAT mutants.

## Introduction

The dopamine transporter (DAT^2^, SLC6A3) terminates synaptic transmission by reuptake of extracellular dopamine into the presynaptic neuron. The transport cycle of DAT is consistent with the alternating access model (1): the transporter presents a substrate-binding site to the extracellular side of the membrane; upon binding of substrate and co-substrates (Na^+^ and Cl^−^), conformational changes occlude the extracellular access pathway and expose the solutes to the intracellular space. This results in their transport into the cell. Subsequently, the transporter returns to the outward facing conformation. This reaction is assumed to be the rate-limiting step of dopamine transport (2). Crystallographic investigations of bacterial transporters, in conjunction with computational modeling, provide compelling evidence in support of this general mechanism (3–5). The crystal structure of the *Drosophila melanogaster* dopamine transporter (dDAT) confirmed that insights from these bacterial SLC6 transporter homologs can be extrapolated to SLC6 neurotransmitter transporters (6).

DAT and its close relatives, *i.e.* the transporters for serotonin and norepinephrine, are targets of several psychoactive substances. These chemically diverse ligands are used for both, therapeutic and recreational purposes, and can be classified with respect to their mode of action on DAT (1). They act as substrates (*e.g.* amphetamines), competitive inhibitors (*e.g.* cocaine), or non-competitive inhibitors (*e.g.* ibogaine), and have in common that they increase extracellular dopamine concentrations because they reduce the net-uptake of dopamine into the cell (1, 7–9).

However there is one notable exception: Zn^2+^, which is an endogenous allosteric modulator of DAT. The mode of Zn^2+^ binding has been elucidated; it binds DAT at an extracellular site, which is coordinated by four residues (10–12). The effect of Zn^2+^ on the function of DAT, however, has remained enigmatic. Both, inhibition and activation of DAT were observed, depending on the experimental assay employed. Originally, Zn^2+^ was found to inhibit substrate uptake (8, 9) but to stimulate amphetamine-induced dopamine release (18). In addition, Zn^2+^ potentiated dopamine-induced currents through DAT. This is inconsistent with reduced substrate influx (11). In fact, Zn^2+^ was subsequently shown to either inhibit or stimulate [^3^H]dopamine uptake in heterologous expression systems, depending on the selected cell line (13). It is evident that these findings are difficult to reconcile.

Here, we examined the hypothesis that Zn^2+^ exerts an allosteric effect on the transport cycle of DAT. Based on our observations, we provide a mechanistic framework, which accounts for all reported actions of Zn^2+^. Most importantly, we show that the kinetics of the transport cycle dictate an intricate coupling between the allosteric effect of Zn^2+^ and the intracellular Na^+^ concentration: at low [Na^+^]*_i_* binding of Zn^2+^ affords accelerated cycling, but at high [Na^+^]*_i_* it acts as a brake.

## Experimental Procedures

### 

#### 

##### Whole-cell Patch-clamp

Patch-clamp recordings were performed with HEK-293 cells stably expressing human DAT (hDAT). The cells were seeded at low density 24 h before recordings. Substrate-induced hDAT currents were recorded under voltage clamp using the whole-cell configuration. Briefly, glass pipettes were filled with a solution consisting of 133 mm K-gluconate, 5.9 mm NaCl, 1 mm CaCl_2_, 0.7 mm MgCl_2_, 10 mm EGTA, and 10 mm HEPES adjusted to pH 7.2 with KOH. Substrate-induced peak currents were isolated using an internal solution comprising 152 mm NaCl, 1 mm CaCl_2_, 0.7 mm MgCl_2_, 10 mm EGTA, and 10 mm HEPES (pH 7.2 with NaOH), which eliminated the steady current component. For some measurements we used a solution containing 50 mm NaCl, 100 mm KCl, 1 mm CaCl_2_, 0.7 mm MgCl_2_, 10 mm EGTA, and 10 mm HEPES (pH 7.2 with KOH).

The cells were continuously superfused with external solution (140 mm NaCl, 3 mm KCl, 2.5 mm CaCl_2_, 2 mm MgCl_2,_ 20 mm glucose, and 10 mm HEPES adjusted to pH 7.4 with NaOH). Currents were recorded at room temperature (20–24 °C) using an Axopatch 200B amplifier and pClamp 10.2 software (MDS Analytical Technologies). Cells were voltage-clamped to a holding potential of −60 mV or 0 mV and the washout period following dopamine application was 30 s in all cases. Current traces were filtered at 1 kHz and digitized at 10 kHz using a Digidata 1320A (MDS Analytical Technologies). Drugs were applied using a DAD-12 (Adams & List, Westbury, NY), which permits complete solution exchange around the cells within 100 ms (14). Current amplitudes in response to application of dopamine or the combination of dopamine and Zn^2+^ were quantified using Clampfit 10.2 software. Passive holding currents were subtracted and the traces were filtered using a 100 Hz digital Gaussian low-pass filter.

##### Radioligand Binding Experiments

Membranes were prepared from a defined number of stably transfected HEK293 cells expressing human DAT. The binding reaction was carried out as described previously (15): briefly, the assay volume was 0.1 ml, the membranes (corresponding to about 1–1.5 × 10^4^* cells/reaction) were incubated in buffer containing 20 mm Tris-HCl, 2 mm MgCl_2_, 120 mm NaCl, and 3 mm KCl, 10 μm ZnCl_2_, and [^3^H]CFT (specific activity: 80 Ci/mmol; 6 to 7 concentrations covering the range of 3 to 120 nm) for 30 min at 20 °C. Nonspecific binding was determined in the presence of 10 μm mazindol.

##### Modeling

The recorded currents were emulated with a previously published kinetic model of the transport cycle of DAT (2). This model was extended to account for binding of Zn^2+^ to DAT. The time-dependent changes in state occupancies were evaluated by numerical integration of the resulting system of differential equations using Systems Biology Toolbox (16) and Matlab 2012a (Mathworks).

The voltage-dependence of individual rates were modeled according to Laeuger (17) assuming a symmetric barrier as *k*_ij_ = *k*0_ij_exp(−zQ_ij_FV/2RT), with F = 96485 Cmol^−1^, *r* = 8.314 JK^−1^ mol^−1^ and V the membrane voltage in volts and T = 293 K. The extra- and intracellular ion concentrations were set to the values used in the experiments. Substrate uptake was modeled as (TiClS × kSioff-TiCl × Si × kSion + TiClSZn × kSioff-TiClZn × Si × kSion) × NC/NA. Where NC is the number of transporters and NA is the Avogadro constant. Currents were simulated assuming a transporter density of 25 × 10^6^/cell.

## Results

### 

#### 

##### Zn^2+^ Causes a Modest Shift in the Concentration-dependence of Dopamine-induced Steady-state Currents through DAT, but Does Not Affect Their Voltage-dependence

**FIGURE 1.**
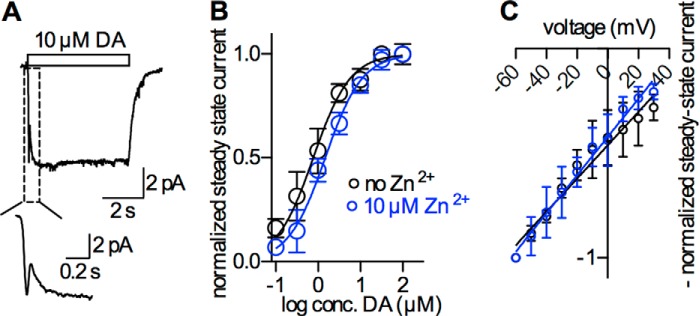
**Concentration-dependent dopamine-induced steady-state currents in the absence and presence of Zn^2+^.** *A*, representative current trace recorded from a HEK-293 cell stably expressing hDAT. Currents were recorded in the whole-cell configuration with the membrane potential clamped to −60 mV. A 5 s application of 10 μm dopamine (*DA*) provoked an inwardly directed current that deactivated after removal of dopamine. The current comprises a peak component (see *inset*) and a steady-state component. *B*, steady-state current as a function of dopamine concentration in the presence (*blue circles*) and absence of 10 μm Zn^2+^ (*black circles*). All currents were normalized to the condition that gave rise to the largest currents. The *solid lines* were drawn by fitting the data points (*n* = 8, error bars = S.D.) to the equation for single binding site. The EC_50_ was 0.77 μm (95% confidence interval: 0.67–0.88 μm) in the absence of Zn^2+^ and 1.48 μm (1.33–1.64 μm) in its presence. The EC_50_ values were significantly different (*p* = 0.001; F-test). *C*, steady-state current as a function of membrane potential. Currents were normalized to the currents at −60 mV, and the respective values were multiplied by −1. The data points were subjected to linear regression;. The slopes of the resulting *blue* and *black line* did not differ in a statistically significant way (*p* = 0.09; F-test).

We analyzed the action of Zn^2+^ on distinct partial reactions of the transport cycle by capitalizing on the high temporal resolution of the whole-cell patch-clamp technique. We selected stably transfected HEK293 cells, which expressed the human DAT to high levels; the number of DAT molecules was estimated in saturation binding experiments of [^3^H]CFT and amounted to 27 ± 3.9 × 10^6^ transporters/cell. Upon rapid substrate application, HEK293 cells expressing hDAT exhibit an inwardly directed current that comprises two components, a transient peak current and a steady-state current (Fig. 1*A*). These currents have been assigned to distinct events in the transport cycle: the peak current has been attributed to the conformational rearrangement that carries substrate through the membrane. The steady-state current is assumed to originate predominantly from the conversion of empty inward facing transporters to the outward facing conformation (2). In other words, the peak current can be used to study substrate translocation, whereas the steady-state current reads out the forward transport mode. Fig. 1*A* shows a typical current elicited by fast application of 10 μm dopamine to a HEK-293 cell stably expressing DAT. The current was measured in the presence of physiological ion gradients, and the membrane potential was clamped to −60 mV. Application of 10 μm dopamine elicited the initial peak current (see *inset* to Fig. 1*A*), which was followed by the sustained steady-state current mentioned above. The steady-state current deactivated upon removal of dopamine from the bath solution.

The magnitude of the steady-state current depended on the concentration of dopamine: *i.e.* it was close to saturation at 30 μm (Fig. 1*B*). The concentration-response curve was adequately described by a saturation hyperbola. The EC_50_ values in the presence and absence of Zn^2+^ were 1.48 μm [1.33 μm-1.64 μm], 0.77 μm [0.67 μm-0.88 μm], respectively.

We verified that Zn^2+^ did not change the current-voltage-relation of the steady-state current through DAT by recording currents between −60 and +30 mV. At potentials more positive than +30 mV, excessive noise precluded further analysis. The currents were induced by application of 30 μm dopamine; the current amplitudes were normalized to the amplitude measured at −60 mV and plotted as a function of voltage: It is evident from Fig. 1*C* that (i) the current-voltage-relation of the dopamine-induced steady-state current was linear over the explored voltage range and (ii) that it was not affected by Zn^2+^.

##### Zn^2+^ Alters the Amplitude and the Kinetics of Currents Associated with Dopamine Transport

**FIGURE 2.**
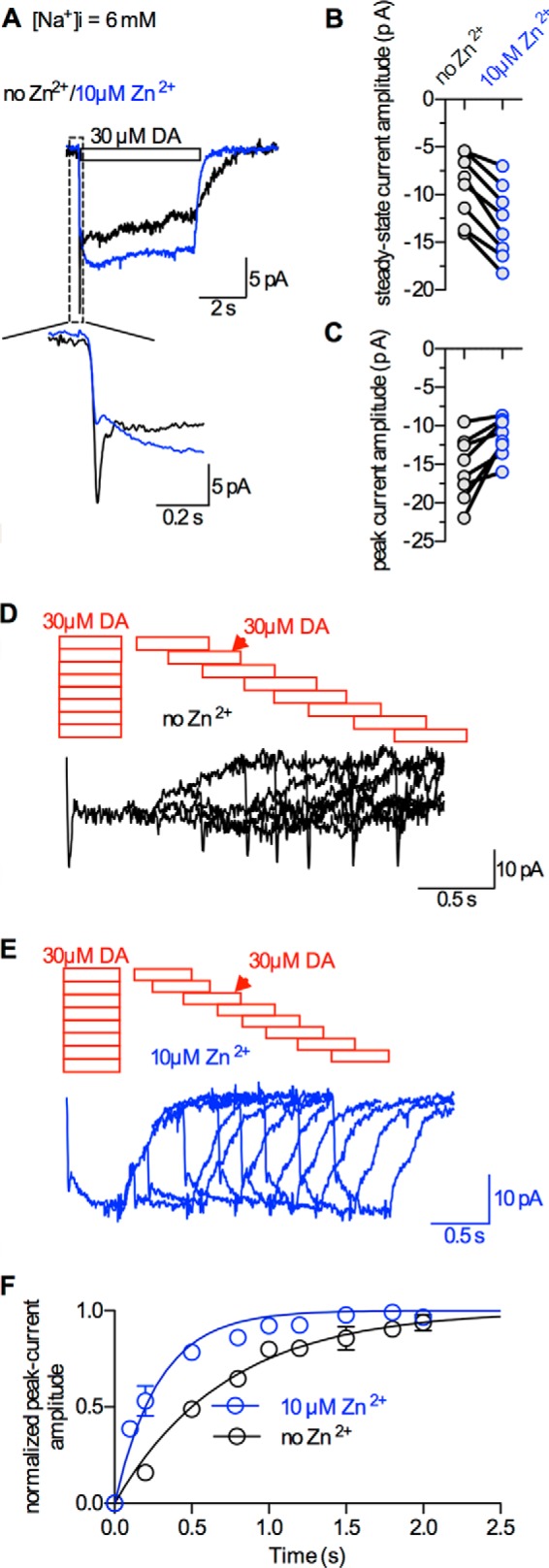
**Zn^2+^ alters the kinetics of currents carried by DAT.** *A*, overlay of two representative current traces recorded from the same cell at −60 mV. *Black trace*: response to 30 μm dopamine (*DA*); *blue trace*: in the presence of 10 μm Zn^2+^. *B* and *C*, steady-state current and the peak current amplitudes, elicited by 30 μm dopamine. The *blue circles* represent values measured in the presence of 10 μm Zn^2+^ and *black circles* in its absence. *Black lines* connect data derived from the same cell. Zn^2+^ (10 μm) potentiated the steady-state current (−9.2 ± 3.5 *versus* −12.0 ± 3.9 pA; *n* = 8, *p* < 0.01, Wilcoxon signed rank test) (*B*), but reduced the peak current (15.5 ± 4.1 *versus* 11.5 ± 2.5 pA; *n* = 8, *p* < 0.01, Wilcoxon signed rank test) (*C*). *D* and *E*, protocol to determine the turnover rate of DAT/recovery after dopamine application and subsequent washout. Dopamine (30 μm) was applied to the cell for 0.5 s, both in the presence (*E*) and in the absence of 10 μm Zn^2+^ (*D*). Subsequently, test pulses of 30 μm dopamine were applied to the cell, 0.1, 0.2, 0.5, 0.8, 1, 1.2, 1.5, and 2 s after dopamine removal. Following dopamine removal, the currents (peak and steady-state) recovered to their initial amplitudes. *F*, recovery of the peak current as a function of time with (*blue circles*) and without 10 μm Zn^2+^ (*black circles*). The peak currents were normalized to the respective largest peak current from the same cell. The data are means ± S.D. (*n* = 6 cells). The data were fit to a mono-exponential function (see *black and blue line*, *R*^2^ = 0.96 and 0.96). The estimated time constants were significantly different: 0.72 s (0.67–0.78 s) (*n* = 7) and 0.29 s (0.26–0.33 s) (*n* = 5) in the absence and presence of 10 μm Zn^2+^ (*p* < 0.0001, F-test), respectively.

Fig. 2*A* shows representative traces of currents induced by 30 μm dopamine in the presence and absence of 10 μm Zn^2+^. Binding of Zn^2+^ to DAT changed the following properties of the dopamine-induced current: (i) it increased the steady-state current (inset to Fig. 2, *A* and *B*) consistent with previous reports, which documented potentiation of dopamine-induced currents by extracellular Zn^2+^ (18, 19) and (ii) decreased the peak current (Fig. 2*C*). (iii) Moreover, the current deactivated more rapidly in the presence of Zn^2+^. The latter finding suggests that the DAT/Zn^2+^ complex relaxes faster to its original state (T_o_NaClZn: see model described below). We tested this conjecture directly by monitoring the time course of peak current recovery after dopamine application and subsequent washout. For these measurements, we exploited the fact that the peak current amplitude is proportional to the number of transporters available for dopamine binding. Thus, the peak current amplitude provides a read-out for occupancy of the outward facing conformation (T_o_NaCl and T_o_NaClZn). Similarly, the time course of peak current recovery allows for estimating the time required for the transporter to return to the outward facing conformation. We applied 30 μm dopamine for 0.5 s and subsequently removed dopamine from the bath solution for 0.1, 0.2, 0.5, 0.8, 1, 1.2, 1.5, and 2 s, respectively. We then re-applied 30 μm dopamine to determine the fraction of transporters that had returned to T_o_NaCl/T_o_NaClZn and were therefore available to support another peak current. It is evident from Fig. 2, *D* and *E* that peak current amplitudes increased in magnitude as a function of the dopamine-free interval. In Fig. 2*F*, this time course of recovery is plotted in the presence and absence of 10 μm Zn^2+^: the data were well described by a mono-exponential function. The fit yielded time constants of 0.29 s (0.26–0.33 s) and 0.72 s (0.67–0.78 s) in the presence and absence of Zn^2+^. The time course of recovery is a measure of the turnover rate of DAT, *i.e.* the time needed to complete one conformational cycle. Thus, the analysis yields a turnover rate of DAT in the absence of Zn^2+^ of 1.45 s^−1^ (*versus* 3.45 s^−1^ in the presence of Zn^2+^). This rate agrees well with earlier estimates (2, 20). The observations therefore suggest that the turnover rate of DAT is larger in the Zn^2+^-bound state. This interpretation, however, disagrees with studies, which documented inhibition of substrate uptake by Zn^2+^ (11, 12).

##### The Action of Zn^2+^ on DAT Depends on the Intracellular Na^+^ Concentration

**FIGURE 3.**
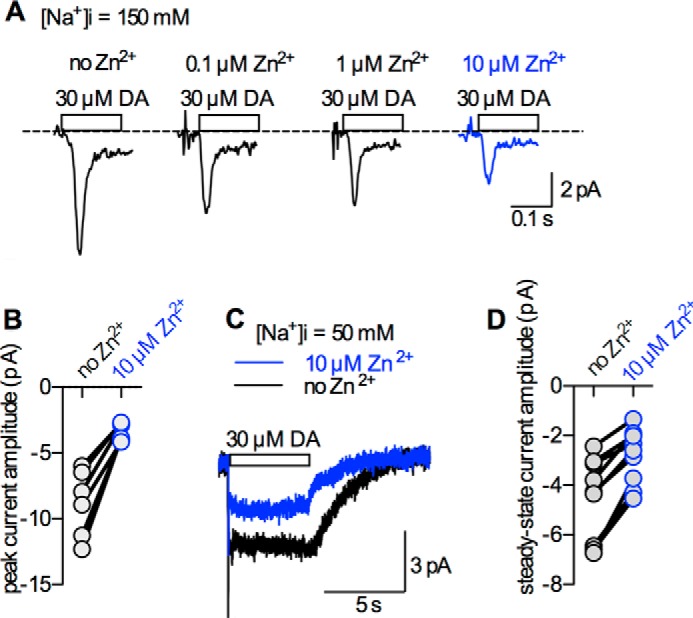
**The action of Zn^2+^ on hDAT depends on [Na^+^]_i_.** *A*, representative current traces of dopamine-induced (DA, 30 μm) currents by DAT expressed in HEK-293 cells. Currents were measured at 0 mV and in the presence of 150 mm [Na^+^]*_i_*. The latter eliminates the steady state current but keeps the peak current intact. Zn^2+^ reduced the peak current amplitude in a concentration-dependent manner (0.1, 1, and 10 μm). *B*, *black circles*: peak current amplitudes in the absence of Zn^2+^; *blue circles*: respective values in the presence of 10 μm Zn^2+^. Zn^2+^ significantly reduced the peak current amplitudes (−8.8 ± 2.6 *versus* −3.4 ± 0.7 pA; *n* = 6, *p* < 0.05, Wilcoxon signed rank test). *C*, overlay of representative current traces induced by 30 μm dopamine in the presence (*blue trace*) and absence (*black trace*) of 10 μm Zn^2+^ both recorded at −60 mV. The cells contained 50 mm [Na^+^]*_i_*. This reduced the steady-state current amplitude but kept it at a measureable level. At this [Na^+^]*_i_*, 10 μm Zn^2+^ inhibited the steady-state current. *D*, *black* and *blue circles* represent the steady-state current amplitudes measured in the absence and presence of 10 μm Zn^2+^, respectively. Steady state current amplitudes were significantly reduced (−4.5 ± 1.7 *versus* −2.8 ± 1.1 pA; *n* = 8, *p* < 0.01, Wilcoxon signed rank test).

The analysis of the Zn^2+^ action on the peak current by DAT is hampered by the confounding presence of the steady-state current. Therefore, we measured the peak current in the absence of the steady-state current. This can be achieved by using high intracellular Na^+^ concentrations ([Na^+^]_i_: 150 mm) (2, 21). An intracellular concentration of 150 mm Na^+^ favors rebinding of Na^+^ to the inward facing transporter, thus precluding progression through the transport cycle. This eliminates the steady-state current, but keeps the peak current intact. Fig. 3*A* shows typical current traces. Shown are representative peak currents elicited by 30 μm dopamine measured at 0 mV before and after application of 0.1, 1, and 10 μm Zn^2+^. Zn^2+^ reduced the peak current amplitude. The magnitude of the effect is quantified for 6 independent recordings on different cells in Fig. 3*B*. Importantly, the inhibition by Zn^2+^ exceeded that seen in the measurements summarized in Fig. 2*C*: when the currents were measured in the presence of 6 mm Na^+^_i_, 10 μm Zn^2+^ reduced the peak current by only 30%. In contrast at 150 mm Na^+^_i_, 10 μm Zn^2+^ reduced the peak by ∼70%. This suggests that Na^+^_i_ rendered the process that gives rise to the peak current more sensitive to Zn^2+^. We then investigated whether Na^+^_i_ also alters the Zn^2+^ action on the steady-state current. For this purpose, we set [Na^+^]*_i_* to 50 mm. As predicted, the steady-state current was lower at 50 mm Na^+^_i_ than at 6 mm Na^+^_i_, because the progress through the cycle was impeded. More importantly, however, under these conditions, 10 μm Zn^2+^ led to a reduction of the steady-state current (Fig. 3, *C* and *D*). This contrasts with the observed current potentiation at 6 mm Na^+^_i_ (see Fig. 2, *A* and *B*).

##### Zn^2+^ Dissociation from DAT Is State-dependent

**FIGURE 4.**
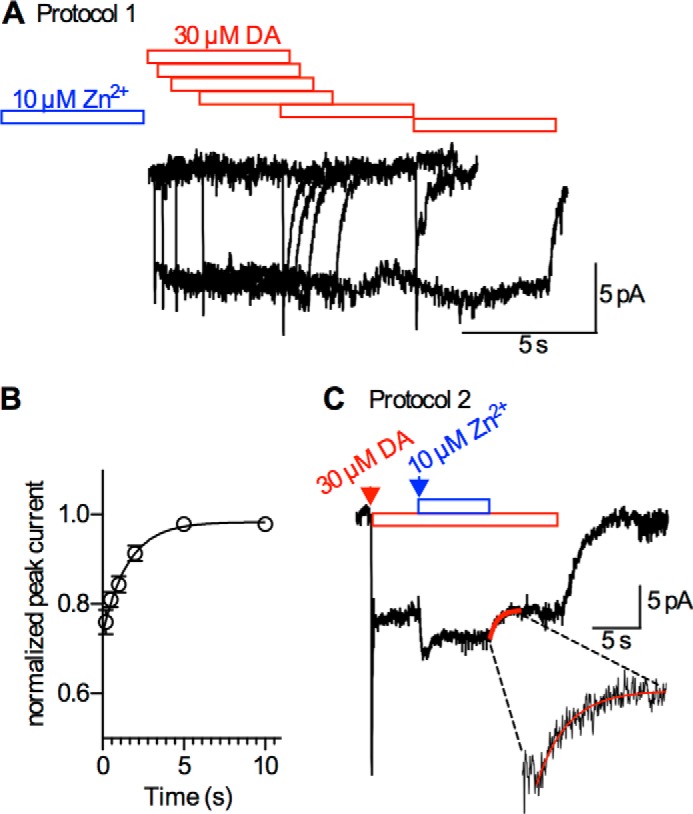
**Measurements of the dissociation rate of Zn^2+^ from the outward (protocol 1) and the inward facing conformation (protocol 2).** *A*, protocol 1: a HEK293 cell stably expressing hDAT was clamped to −60 mV and exposed to 10 μm Zn^2+^ for 5 s. Following a Zn^2+^-free interval of 0.2, 0.5, 1, 2, 5, and 10 s, respectively, the cell was challenged with 30 μm dopamine for 5 s. Application of dopamine elicited a current comprised of a peak and a steady current component. The peak current was suppressed at the first time point (0.2 s after removal of Zn^2+^). This suppression gradually vanished as the Zn^2+^-free interval was prolonged. *B*, normalized peak currents as a function of the duration of the Zn^2+^-free interval. The *straight line* indicates a fit of a mono-exponential function to the data-points (*n* = 6). The time-constant for the peak current recovery as estimated by the fit was 1.57s (95% confidence interval: 1.06–3.01 s). *C*, protocol 2: a HEK293 cell expressing DAT was clamped to −60 mV and challenged with 30 μm dopamine. Dopamine induced a peak and a steady-state current. The latter increased upon Zn^2+^ co-application (10 μm). Upon removal of Zn^2+^ the current relaxed to its prior level. The *inset* shows the time-course of current relaxation upon Zn^2+^ removal. The *red straight line* indicates a fit of mono-exponential function to the current trace. The derived time constant from six independent measurements was 0.21 s (±0.06 s).

It has been proposed that Zn^2+^ binds to the outward facing state of DAT with higher affinity than to the inward facing state (10, 22). To verify this conjecture by an experimental approach, we designed a protocol to measure the dissociation rate of Zn^2+^ from the outward facing conformation (Fig. 4*A*) and a complementary protocol (Fig. 4*C*) for the inward facing conformation. For the former, we relied on peak current suppression by Zn^2+^ (see Fig. 2), which is expected to vanish upon Zn^2+^ dissociation. In the protocol depicted in Fig. 4*A*, we exposed cells to Zn^2+^ for 5 s and subsequently measured the recovery of the peak current as a function of the time interval of Zn^2+^ washout. The time course of peak current recovery was adequately described by mono-exponential rise (Fig. 4*B*) and thus provided a measure of the dissociation rate of Zn^2+^ (τ = 1.57 s). However, the modest peak current reduction by Zn^2+^ (∼20–30%) limited the dynamic range of this measurement. This is reflected by the large 95% confidence interval for the time constant estimated by the fit 1.57s (1.06–3.01 s). These measurements were conducted with physiological ion gradients in place, a condition at which DAT was shown to adopt the outward facing conformation. Hence, the measured rate most likely represents the dissociation rate of Zn^2+^ from the outward facing conformation.

The other protocol to measure Zn^2+^ dissociation is shown in Fig. 4*C*. Here, we first applied 30 μm dopamine to induce a current. After 5 s, 10 μm Zn^2+^ was co-applied; this led to the expected potentiation of the steady-state current. Subsequently, Zn^2+^ was removed from the solution and the steady-state current relaxed to its initial amplitude. The time course of this relaxation is another measure of Zn^2+^ dissociation (Fig. 4*C*, *inset*) and it was well described by a mono-exponential function. The estimated time constant for Zn^2+^ dissociation under this condition was 0.21 s ± 0.06 s (Fig. 4*C*). Thus, this time constant (for relaxation of steady-state current) is ∼10 times smaller than the one obtained by measuring recovery of peak current. This second protocol differs considerably from the first, because upon dopamine binding, DAT enters the transport cycle. Accordingly, under this condition, the transporter visits all conformations within the transport cycle, including those that are inward facing. The faster rate of Zn^2+^ dissociation observed with the latter protocol is therefore consistent with the idea that Zn^2+^ dissociates faster from the inward facing conformation of DAT.

It is evident that the dopamine test pulses employed in the first protocol (peak current recovery) elicit currents that display a mixed response: while the peak current suppression by Zn^2+^ was still observed in test pulses following a Zn^2+^ free interval of 2 s, the two other effects by Zn^2+^ (current potentiation and accelerated current deactivation) were not observed in any of the test pulses including those following the shortest Zn^2+^ free interval (0.2 s, see Fig. 4*A*). This differential effect is consistent with the fast Zn^2+^ dissociation observed in the second protocol, *i.e.* under the condition, where DAT is allowed to undergo the transport cycle to generate the steady state current.

##### Effect of Zn^2+^ on Dopamine-induced Membrane Depolarization

**FIGURE 5.**
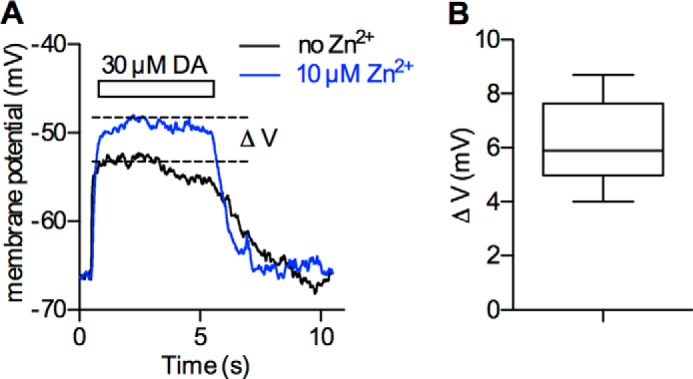
**Effect of Zn^2+^ on dopamine-induced membrane-depolarization.** *A*, overlay of two recordings from a HEK-293 cell stably expressing DAT measured in the current clamp configuration. The traces show the membrane voltage over time. Application of 30 μm dopamine (*DA*, *black trace*) to the cell for 5 s led to a depolarization of the cell membrane that was more pronounced in the presence of 10 μm Zn^2+^ (*blue trace*). *B*, difference in depolarization (ΔV) in the absence and presence of 10 μm Zn^2+^ (6.2 ± 1.6 mV, *n* = 6).

Meinild and coworkers showed that application of dopamine together with Zn^2+^ gave rise to a larger depolarization than dopamine alone when the membrane voltage was measured in unclamped *Xenopus laevis* oocytes (18). We reproduced this finding in unclamped HEK293 cells stably expressing DAT. However, the average difference in voltage (V_DA&Zn_^2+^ − V_DA_) was only ∼6 mV (Fig. 5, *A* and *B*). This may contribute to the reported inhibitory action of Zn^2+^ on cellular substrate uptake, but it is unlikely to account for its reported magnitude (*i.e.* by 50%). Given our measured current-voltage-relation (see Fig. 1*C*), a change by 6 mV is predicted to only modestly affect substrate uptake by DAT.

## Discussion

**FIGURE 6.**
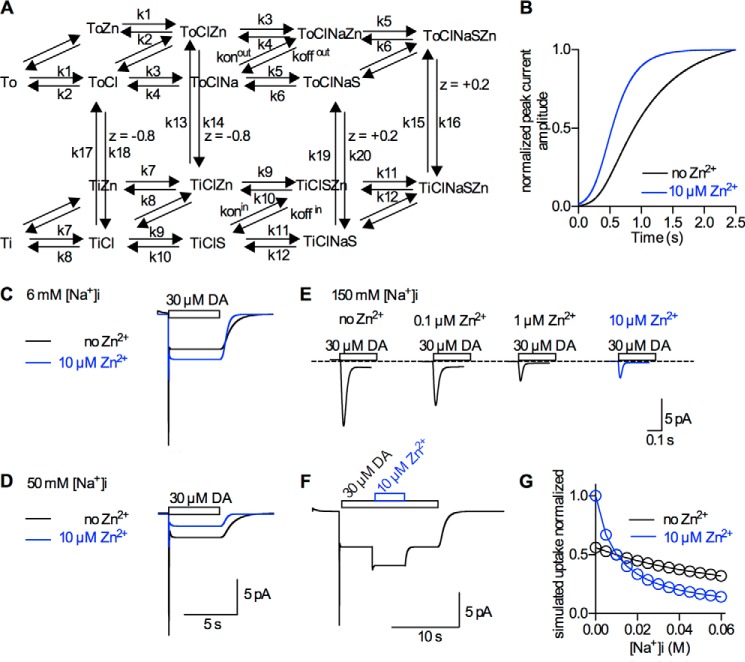
**A kinetic model for the Zn_2+_ action on DAT.** *A*, scheme of the transport cycle of DAT based on the model proposed by Erreger *et al.* (2). To account for Zn^2+^ binding, we added a Zn^2+^-bound state to each state in the original scheme. To account for the stabilization of the outward facing conformation of DAT by Zn^2+^, we assumed a 10-fold higher affinity for all outward-facing states (*k*_on_^out^ = 1 × 10^7^, *k*_off_^out^ = 0.5, *k*_on_^in^ = 1 × 10^7^, *k*_off_^in^ = 5). The rates of the model were adopted according to the data gathered in the present study. The rates were as follows (s^−1^/Mol^−1^ × s^−1^): *k*1 = 5 × 10^5^, *k*2 = 2000, *k*3 = 10^4^, *k*4 = 100, *k*5 = 10^7^, *k*6 = 10, *k*7 = 5 × 10^6^, *k*8 = 200, *k*9 = 4 × 10^7^, *k*10 = 40, *k*11 = 10^7^, *k*12 = 10^5^, *k*13 = 1, *k*14 = 0.1, *k*15 = 500, *k*16 = 50, *k*17 = 0.5, *k*18 = 5, *k*19 = 5, *k*20 = 50. *B*, simulated peak current recovery after dopamine application assuming 2.5 × 10^7^ DAT molecules/cell. This measure of the turnover rate of DAT agrees well with the data in Fig. 2*F. C–E*, simulated currents at 6, 50, and 150 mm [Na^+^]*_i_* with/without Zn^2+^. The simulated traces match the experimental data shown in Figs. 2*A*, 3*A*, and 3*C*, respectively. *F*, simulated currents elicited by the protocol to measure Zn^2+^ dissociation from the inward facing conformation of DAT. The simulation matches the data in Fig. 4*C. G*, simulated substrate uptake as a function of [Na^+^]*_i_*. The model predicts enhanced substrate uptake by Zn^2+^ below 10 mm [Na^+^]*_i_* and inhibition of substrate uptake above 10 mm [Na^+^]*_i_*.

DAT is endowed with an endogenous Zn^2+^-binding site, which is absent in the closely related transporters for the other monoamines (10–12). In the original description of the Zn^2+^ action on substrate transport by DAT expressed in HEK293 cells, 10 μm Zn^2+^ decreased uptake of dopamine by approx. 50% (12). Based on this finding, Zn^2+^ has been suggested to act as inhibitor of DAT by restricting a conformational change required for the transport process. This inhibitory action has been reproduced by others (10, 23). Nevertheless, there are other reports, which showed stimulation of substrate uptake by Zn^2+^ or failed to detect an inhibition (13, 18, 24): A notable example is a study conducted by Pifl *et al.* where the nature of the Zn^2+^ action depended on the expression system. While Zn^2+^ suppressed substrate uptake in HEK-293 cells expressing DAT, it stimulated transport when DAT was expressed in SK-N-MC cells (13). Moreover, in voltage-clamped *X. laevis* oocytes, Zn^2+^ did not cause any change in substrate uptake by DAT (18). It is unsatisfactory to ascribe these discrepancies to variation in the experimental assay and/or cell type employed without a rational explanation. The current experiments were designed to explore the mechanistic basis for the action of Zn^2+^ by exploiting the high temporal resolution provided by electrophysiological recordings. The precision of the recordings is adequate to justify their incorporation into a kinetic model (Fig. 6*A*), which is based on the model proposed by Erreger *et al.* (2): the original model related the two types of currents, which were recorded in the present study, to the transport cycle of DAT. Here, we show that Zn^2+^ affects both current components, *i.e.* the peak current reflecting the substrate translocation step and the steady state-current, which was suggested to originate predominantly from the return of the empty transporter. In agreement with previous reports, we found potentiation of the steady-state current (18, 19). In addition, we observed that Zn^2+^ reduces the amplitude of the dopamine-induced peak current. Finally, Zn^2+^ has been suggested to stabilize DAT in the outward facing conformation (22). Stabilization of the outward facing conformation by Zn^2+^ implies that the latter binds Zn^2+^ with higher affinity than the inward facing conformation. Our experiments examined this conjecture and found two dissociation rates of Zn^2+^, which differed approximately by a factor of ten.

The current kinetic model accounts for Zn^2+^ binding by adding a Zn^2+^-bound state to each state in the original scheme. (see resulting scheme, Fig. 6*A*). Based on our observations, the affinity of Zn^2+^ for the outward facing conformation was assumed to be 10-fold higher than for the inward facing conformation (see Fig. 4). The differing affinities required an adjustment in the rates of two reaction steps of the transport cycle to maintain microscopic reversibility. The reactions that were inevitably affected by these manipulations were the following: (i) conversion of the empty inward facing transporter to the outward facing conformation (*i.e.* the rate-limiting step) and (ii) isomerization of substrate-loaded transporters from the inward to outward facing conformation (*i.e.* the substrate exchange reaction).

The model evidently accounts for the observed changes in the dopamine-induced currents that occur upon Zn^2+^ binding (Fig. 6*C*). In the model, Zn^2+^ potentiates the steady-state current because the rate-limiting step in the transport cycle of DAT, *i.e.* the return step, is accelerated. Consequently, one prediction of the model is that Zn^2+^ increases the efficacy of substrate transport by DAT. In light of this finding, Zn^2+^ can be considered as an activator of transport. This conclusion is at odds with the reported inhibitory effect of Zn^2+^ on substrate uptake, but fits with a previous study that showed stimulation of uptake by Zn^2+^(12, 13). The model suggests that the peak current amplitude is reduced by Zn^2+^, because a sizeable fraction of transporters, which converted from the outward to inward facing conformation with dopamine bound, returns immediately. The returning fraction of transporters carries the charge that normally gives rise to the inwardly directed peak current into the opposite direction. As a consequence, the net-charge and hence the peak current amplitude is reduced.

Importantly, the model predicts that Zn^2+^ either increases or decreases substrate uptake by DAT, depending on the [Na^+^]*_i_*. This can be explained as follows: Zn^2+^ enhances the conversion from the inward to the outward facing conformation regardless of whether the transporter is empty or substrate loaded. High [Na^+^]*_i_* prolong the dwell-time in the substrate-bound form; thus, under this condition, Zn^2+^ will favor the substrate-exchange mode of the transporter. This leads to inhibition of uptake. Conversely, low [Na^+^]*_i_* (*e.g.* 6 mm) is predicted to permit completion of the transport cycle. Given that Zn^2+^ accelerates the rate-limiting step, this must result in enhanced uptake.

Several lines of evidence support this notion: (i) if 150 mm Na^+^_i_ is applied internally (a condition that fully eliminates the steady-state current) the peak current is rendered more sensitive to the inhibition by Zn^2+^ (ii) at 50 mm Na^+^_i_, a condition at which the steady-state current is reduced but not fully eliminated, Zn^2+^ suppresses the dopamine-induced steady-state current. The latter finding contrasts with the observed potentiation of the steady-state current by Zn^2+^ at low [Na^+^]*_i_* (6 mm). (iii) Larger currents with Zn^2+^ at low [Na^+^]*_i_*, on the other hand, are consistent with the hypothesis that Zn^2+^ can enhance substrate uptake. This is further supported (*iv*) by our measurement of the turnover rate of DAT, which was greater in the presence of Zn^2+^. It is worth pointing out that our model can faithfully reproduce these observations (*see* Fig. 6, *B–E*). In addition, the model does emulate the currents obtained by the protocol employed to measure Zn^2+^ dissociation from the inward facing conformation with amazing fidelity (Fig. 6*F*). Moreover, we show in Fig. 6*G* the simulated dependence of substrate uptake on [Na^+^]*_i_*. At ∼10 mm [Na^+^]*_i_*, the effect of Zn^2+^ on uptake is predicted to switch from stimulatory to inhibitory.

Our observations and our model provide a plausible explanation for the contradicting reports on the Zn^2+^ action on DAT. We surmise that in those studies, where Zn^2+^ exhibited an inhibitory activity, [Na^+^]*_i_* was high: [Na^+^]*_i_* accumulation is *per se* a consequence of heterologous overexpression of DAT in non-excitable cells such as HEK-293 cells, because (in contrast to neurons) they lack the capacity to rapidly clear the influx of Na^+^. This view is supported by measurements of [Na^+^]*_i_*: Khoshbouei *et al.* demonstrated that, upon application of dopamine to DAT-expressing HEK-293 cells, [Na^+^]*_i_* ramped up to ∼50 mm within a time period of 60 s (25). This is the concentration, where we observed inhibition of the transport cycle by Zn^2+^. In addition, it is the minimum time interval over which cellular uptake assays are conducted.

The study by Meinild *et al.* invoked an uncoupled Cl^−^ conductance as a cause for the larger depolarization in the presence of Zn^2+^ (18). However, our measurements of currents carried by wild-type DAT do not support this interpretation: if Zn^2+^ had also triggered a Cl^−^ conductance, it should have altered the voltage dependence of the steady-state current. Specifically, Zn^2+^ should have shifted the reversal potential to more negative voltages. This, however, was not the case. Therefore we surmise that the Cl^−^ conductance described by Meinild *et al.* is most likely a property of the mutant DAT Y335A, which the authors had used to conduct their analysis. Zn^2+^ has also been shown to inhibit uptake of [^36^Cl^−^] by DAT-expressing HEK-293 cells (13). Our kinetic model accounts for this finding because Cl^−^ uptake is limited when the transporter is trapped in the exchange mode. Hence, we consider this effect collateral rather then causative. In this context it is also worth pointing out that our model can explain why Zn^2+^ enhances substrate release by DAT (23). The exchange mode is a prerequisite for substrate release (9, 26, 27). In accordance, if Zn^2+^ binding favors substrate exchange, Zn^2+^ is predicted to increase amphetamine-induced substrate release.

The stimulatory action of Zn^2+^ on DAT may be of clinical relevance. The monoamine transporters are prominent targets of several therapeutic drugs. To date, all of these drugs are either inhibitors or substrates. Here, we show that an allosteric modulator like Zn^2+^ can act as an activator of substrate uptake when DAT works in the forward transport mode. This novel type of pharmacological action is therapeutically desirable to treat loss-of function mutations of DAT (28). For example, recently, a *de novo* mutation of DAT associated with attention deficit hyperactivity disorder (ADHD) was shown to exhibit reduced activity in the forward and reverse transport mode (29). Most importantly, Zn^2+^ restored the function of this mutation. This is consistent with the present model: Zn^2+^ fosters the forward and reverse operating mode of DAT; thus, it is predicted to restore its function. It is therefore conceivable that a small diffusible compound could emulate the action of Zn^2+^ and could be used as treatment for such disorders.

## Author Contributions

Y. L., P. S. H., K. S., H. H. S., M. F., and W. S. conceived the study. Y. L. and P. S. H. conducted experiments. Y. L. and W. S. analyzed the data. P. S. H., K. S., and W. S. designed the model. Y. L., P. S. H., M. F., and W. S. wrote the manuscript. All authors reviewed the results and approved the final version of the manuscript.

## Footnotes

2 The abbreviations used are:

DAT: dopamine transporter
ADHD: attention deficit hyperactivity disorder
HEK-293 cells: human embryonic kidney cells 293.
